# Integration without decoupling? Hybrid governance and systemic equity in the proposed NBA–FIBA European League

**DOI:** 10.3389/fspor.2026.1841860

**Published:** 2026-07-27

**Authors:** Dimitrios I. Bourdas, Panteleimon Bakirtzoglou, Antonios K. Travlos, Apostolos Theos

**Affiliations:** 1 Section of Sport Medicine & Biology of Exercise, School of Physical Education and Sports Science, National and Kapodistrian University of Athens, Daphni, Greece; 2 School of Physical Education and Sport Science, Aristotle University of Thessaloniki, Thessaloniki, Greece; 3 Department of Sports Organization and Management, Faculty of Human Movement and Quality of Life Sciences, University of the Peloponnese, Sparta, Greece; 4 Section of Sports Medicine, Department of Community Medicine and Rehabilitation, Umeå University/Umeå School of Sport Sciences, Umeå University, Umeå, Sweden

**Keywords:** competitive balance and redistribution, environmental sustainability in sport, franchise vs. promotion-relegation systems, hybrid sport governance, performance ecology, sport analytics and data governance, supporter culture and institutional embeddedness, transnational league integration

## Highlights

NBA-Europe represents a natural experiment in hybrid sport governance, embedding franchise-based commercial logics within Europe's meritocratic, pyramid-structured basketball ecosystem. This convergence reconfigures performance ecology, coaching authority, redistribution mechanisms, and stakeholder legitimacy across interconnected institutional levels.

The central analytical tension concerns integration without decoupling: whether commercial consolidation, analytics intensification, and transnational expansion can coexist with competitive permeability, developmental pluralism, supporter embeddedness, and ecological accountability.

The long-term viability of the NBA–FIBA joint venture will depend not on financial growth alone, but on sustaining systemic equity and reciprocal embeddedness across governance, performance, and cultural domains.

## Introduction: transatlantic hybrid governance in European basketball

The European basketball ecosystem is entering a phase of structural reconfiguration following the announced collaboration between the National Basketball Association (NBA) and the International Basketball Federation (FIBA) (1). Targeted for launch in October 2027, NBA-Europe represents not simply expansion but a deliberate governance intervention: the embedding of a closed-franchise architecture—defined by centralised authority, revenue sharing, collective bargaining, and salary-cap regulation—within Europe's historically pluralistic, promotion–relegation-based sport system (2). The initiative therefore constitutes a contested process of transatlantic governance convergence rather than neutral market development.

European professional basketball has long operated within a bifurcated structure characterised by tension between shareholder-driven EuroLeague commercial autonomy and FIBA's pyramidal, redistribution-orientated legitimacy. The proposed 16-team league—comprising 12 permanent franchises in major metropolitan hubs and four merit-based qualification slots linked to the Basketball Champions League (BCL) (3)—attempts to mediate these competing institutional logics without fully resolving their underlying incompatibilities. By coupling fixed franchise valuation (4) with limited performance-based permeability, the model stabilises capital flows while retaining only partial alignment with European sporting norms (2, 5, 6).

This synthesis introduces a structural asymmetry between regulatory form and managerial rationality. While competition would remain under FIBA's 40-minute format and spatial regulations, managerial incentives are likely to align with NBA-derived performance logics: pace-and-space optimisation, star-centred commodification, data-integrated load management, and advanced biometric infrastructures (7). Comparable processes of tactical diffusion have accompanied previous phases of basketball globalisation, where NBA offensive concepts have progressively influenced European professional competitions while remaining partially constrained by FIBA regulations. In effect, governance appears hybrid, but performance rationality may progressively converge toward franchise-centred efficiency imperatives.

From a sports science and coaching perspective, such convergence is unlikely to be neutral. It recalibrates tactical priorities, physiological benchmarks, and developmental trajectories. Athletes face intensified schedule density, expanded biometric surveillance, and algorithmic performance profiling (8). Coaching authority may shift from intuition-dominant hierarchies toward analytics-integrated, interdisciplinary decision systems. At the ecosystem level, franchise stabilisation places structural pressure on Europe's club-embedded academy model, historically orientated toward long-term formation and solidarity-based redistribution (9).

This article advances the position that the central challenge of NBA-Europe is not integration *per se*, but integration without systemic decoupling. Drawing selectively on institutional logics and systems thinking (10, 11), the NBA–FIBA initiative is interpreted as a hybrid governance formation in which competing rationalities—franchise centralisation and pyramid-based redistribution—interact, overlap, and risk partial decoupling across interconnected domains. European basketball is thus approached as an interdependent system (12, 13) in which changes in governance cascade into performance structures, labour organisation, developmental pathways, and stakeholder legitimacy.

Given the initiative's recency, this opinion article advances a theoretically informed analytical perspective rather than empirical claims. Rather than providing an exhaustive review of each governance domain, it offers a selective systems-level interpretation of those dimensions that are most likely to interact under NBA–FIBA hybridisation. The objective is not to examine each domain in isolation, but to illustrate how tactical, physiological, organisational, developmental, environmental, and governance dynamics become mutually reinforcing within a single institutional transformation. While additional dimensions warrant future inquiry, the present article argues that hybridisation will generate uneven adaptation rather than seamless convergence, and explores whether institutional integration can be sustained without eroding redistribution, competitive permeability, and sociocultural embeddedness. In parallel, the article delineates a focused research agenda to empirically track this transformation, highlighting key domains—workload regulation, competitive concentration, technological asymmetries, environmental externalities, and supporter identity adaptation—through which the consequences of hybrid governance can be systematically assessed.

## Tactical reconfiguration under hybrid regulatory conditions

From the perspective of integration without decoupling, tactical adaptation represents the first arena in which hybrid governance may generate either productive convergence or structural tension. The interaction between NBA and FIBA regulatory logics extends beyond stylistic variation to a structural collision between two historically embedded tactical systems shaped by different institutional incentives. NBA governance has incentivised revenue-maximising aesthetics—high possession volume, spatial expansion, and star-centred isolation sequences that enhance commodification (14). By contrast, FIBA competitions have evolved around spatial density, collective defensive synchronisation, and possession-level patience (6). In this opinion, convergence should be understood not as stylistic blending but as a forced recalibration of spatial organisation, tempo governance, and decision hierarchies under competing performance rationalities.

A principal axis of divergence concerns space–time constraints. The NBA Defensive Three-Second Rule expands interior driving lanes (15), whereas FIBA's regulatory structure—combined with smaller court dimensions—produces interior congestion (16). This divergence is not trivial: it fundamentally reshapes perceptual–motor demands and biomechanical loading patterns. If NBA managerial logic elevates pace within this compressed geometry, transition frequency may interact with reduced displacement margins to intensify eccentric loading and cumulative fatigue (2, 17). The likely outcome is a structural paradox: offensive expansion pursued within a defensive architecture that systematically rewards spatial compression.

A second divergence concerns the tension between analytical efficiency and regulatory elasticity. NBA shot-selection doctrine privileges restricted-area attempts and corner threes (18), yet its translation into the FIBA context is non-linear (19). Recent analyses already document increasing three-point volume and pace across several European elite competitions (20, 21), illustrating that tactical convergence is an ongoing rather than hypothetical process, although important contextual differences remain. Under FIBA rules, post-rim contestability increases the strategic value of second-chance actions and interior timing (16, 22). This modifies expected-value hierarchies, suggesting that direct transplantation of NBA efficiency models may be analytically reductive rather than optimal.

Game duration further alters probabilistic structure. The 40-minute format compresses possessions, increasing the marginal cost of inefficiency. In this context, high-variance isolation strategies may become structurally suboptimal, particularly in late-game scenarios where marginal gains are decisive.

Beyond rule mechanics, centralised governance introduces the risk of strategic homogenisation. European competitions have historically exhibited stylistic plurality rooted in coaching traditions, whereas NBA-affiliated systems trend toward convergence (7). The central tension, therefore, is whether hybridisation will sustain pluralistic tactical identities or progressively standardise decision-making around a single efficiency-dominant paradigm, thereby signalling early-stage decoupling between European tactical heritage and franchise-driven optimisation.

## Biometric governance and workload standardization in high-density competition

The physiological consequences of hybrid governance extend beyond workload alone to the governance of athlete performance within a substantially denser competitive ecology. The adoption of NBA managerial logic would redefine—not merely adjust—the physiological contract between athlete and organisation. European basketball has traditionally operated within high-intensity yet comparatively lower-volume schedules, whereas the NBA model prioritises sustained exposure, dense travel, and continuous monitoring (23). This shift should be interpreted as a transition from intensity-dominant preparation to surveillance-mediated volume governance.

Although FIBA games are shorter, physiological density remains high, with players frequently operating near maximal thresholds (24–26). Introducing an NBA-style schedule creates a volume-intensity paradox: maintaining high per-possession demands while expanding total exposure. Empirical evidence links congested schedules to fatigue accumulation and neuromuscular disruption (27–29). From an opinion standpoint, the critical issue is not whether load increases but whether existing European periodisation models are structurally compatible with such escalation.

Deceleration load further complicates adaptation (30). High-intensity braking actions frequently exceed accelerations across positions in elite European competition (31). If transition-orientated principles are imposed within compressed spatial geometry, eccentric stress may intensify (32). Absent systematic integration of tendon-capacity development, this may produce predictable increases in overuse pathology rather than isolated injury events (30).

Technological integration will accelerate. Wearable systems and machine-learning models enable individualised workload profiling (7). While these tools enhance diagnostic precision (33), it seems reasonable to assume that their uncritical adoption risks epistemological narrowing: privileging quantification over contextual interpretation. Monitoring, in itself, does not mitigate risk; it must be embedded within adaptive conditioning frameworks.

Travel-related circadian disruption may introduce an additional systemic stressor. Sleep quality has demonstrable links to technical efficiency (34–36). Thus, expanded geographic scheduling may degrade performance not through acute fatigue, but through cumulative micro-impairments in perceptual precision.

Simultaneously, intensified biometric governance raises questions of data ownership and autonomy (37). Here, hybrid governance intersects directly with European labour norms, introducing potential regulatory friction beyond performance science.

The biometric transformation implied by NBA-Europe is institutional rather than technological, reconfiguring periodisation, recovery governance, and data rights within a substantially denser competitive ecology. The governing question is whether this model can sustain physiological resilience and institutional legitimacy simultaneously—or whether escalating exposure and surveillance will induce systemic strain.

## Coaching authority and analytics integration in hybrid governance

Hybrid governance also redefines coaching legitimacy by redistributing authority across data, organisational structures, and professional expertise. The implementation of an NBA-managed league would restructure coaching authority from individual command toward network-embedded decision-making. The traditional European model is grounded in centralised control, where the head coach defines tactical, developmental, and disciplinary frameworks (38, 39). By contrast, the NBA institutionalises distributed governance involving analytics departments, front offices, and performance staff (40, 41). Authority, therefore, becomes relational rather than hierarchical.

This divergence is epistemological. European coaching privileges tacit knowledge and experiential judgement (38, 39), whereas NBA environments embed decision-making within data infrastructures (41, 42). From one viewpoint, this shift represents not augmentation but a redefinition of what constitutes “expertise”. Legitimacy increasingly derives from alignment with data-validated reasoning rather than solely from experiential authority (7, 43–46).

For European coaches, adaptation requires integration rather than replacement. The increasing presence of performance departments, athlete-tracking technologies, and analytics-supported decision systems within elite European basketball (40, 47) suggests that elements of this organisational transition are already emerging, although their extent and long-term implications remain uncertain. Tactical decisions may incorporate real-time biometric indicators and probabilistic models (44, 45). Consequently, the coaching environment transforms into an information-dense system in which judgement is continuously mediated by data inputs (40, 45, 48).

This recalibration carries cultural implications. Coaches whose authority rests on experiential mastery may be required to accommodate algorithmic outputs that contradict intuitive assessment (42, 46, 49). Such moments are not technical adjustments but sites of epistemic tension, where professional identity is actively renegotiated.

Governance restructuring extends to roster construction. Under NBA logic, recruitment is increasingly data-driven and front-office-led (50). This attenuates the traditional European model of coach-led team construction, embedding coaching within broader organisational strategy rather than positioning it as its driver.

Accountability metrics intensify this shift. Advanced models seek to isolate coaching impact (51, 52). The coach thus becomes a measurable variable, subject to continuous evaluation within algorithmic performance systems. In already volatile environments (53), this may accelerate turnover and reduce tolerance for strategic deviation.

A final dimension concerns communication. Data density requires translation. Coaches must integrate physiological, tactical, and psychological information into coherent narratives (54). Authority increasingly depends on interpretive competence rather than directive control (55), reflecting a broader transition from individually exercised authority to organisationally embedded coaching legitimacy. The central question is whether this transformation enhances reflexive professionalism—or erodes the experiential and relational foundations that historically underpinned European coaching effectiveness.

## Developmental embeddedness and academy sustainability under franchise stabilization

Developmental systems constitute perhaps the clearest test of whether franchise stability can coexist with Europe's redistributive basketball model. The integration of a North American franchise architecture (56) into European basketball introduces not only structural change but also a normative tension regarding the future of developmental embeddedness. Europe's academy model operates through a redistributive cycle in which clubs invest in youth infrastructure with the expectation that talent either strengthens the senior team or generates transfer-based revenue that finances subsequent cohorts (57, 58). From one point of view, this financial coupling constitutes the hidden backbone of the European model, binding talent production to economic circulation. Franchise governance risks structurally decoupling these linkages.

Within the traditional framework, academies function simultaneously as developmental institutions and financial assets (57). Mid-tier clubs remain viable through transfer mechanisms that recirculate value across tiers. By contrast, the NBA model internalises player allocation via centralised drafts, eliminating intra-league transfer compensation (56). If replicated, this logic could undermine the economic rationale sustaining independent development hubs. Clubs such as Mega Basket or Joventut Badalona may face declining incentives to maintain capital-intensive academies if downstream value is absorbed within franchise ownership structures.

Current research challenges the notion that the central risk is not simply financial but systemic. Redistribution scholarship identifies inter-tier coupling as a prerequisite for ecosystem sustainability (58, 59). Absent enforceable solidarity mechanisms, hybrid governance may shift from value circulation to value extraction, concentrating capital within licensed franchises and eroding grassroots viability (5, 57).

A parallel concern is developmental homogenisation. European academies traditionally cultivate tactical literacy, adaptability, and positional fluidity (57, 60). Franchise systems, however, tend toward archetype standardisation aligned with commercial visibility. Standardising development around “NBA-ready” prototypes risks narrowing cognitive diversity—one of Europe's comparative advantages.

The proposed merit-based BCL access offers symbolic continuity (61), yet high franchise entry thresholds (5) risk entrenching stratification. This creates what can be conceptualised as a “meritocracy gap”, where formal access exists but practical mobility is financially constrained.

Talent concentration may follow, with the emergence of what may be termed “super-academies”. Comparable patterns of resource stratification have been documented in professional football academy systems, where staffing, infrastructure, monitoring capacity, and developmental provision differ substantially across clubs and academy categories (62, 63). Whether such inequalities would develop into a more concentrated “super-academy” structure under an NBA–FIBA governance model remains an open empirical question. While efficient for elite output, such concentration risks reducing geographic diversity and socio-economic access. Conversely, if—and only if—redistribution is institutionally enforced (1), franchise stability could provide a predictable developmental funding base.

A further dimension concerns sociocultural embeddedness. European basketball remains supporter-based (21, 64) and territorially anchored (65). Based on these premises, the embeddedness could not be peripheral but likely constitutive of development itself, influencing recruitment, identity formation, and performance environments. If franchise logics dilute locality (66, 67), consequences may lead to systemic risks affecting academic enrolment patterns and amplifying competitive imbalances.

Ultimately, the sustainability of the academy model depends less on franchise adoption *per se* than on whether the proposed governance model preserves the coupling between redistribution, development, and local identity.

## Competitive balance, talent concentration, and redistribution equity

Competitive balance provides the principal system-level indicator of whether the proposed governance model preserves equity while pursuing commercial consolidation. In this context, it functions as both a normative benchmark and a structural test of institutional legitimacy. The fusion of franchise-based revenue concentration with open European competition raises questions not only of parity but also of systemic legitimacy.

Economic theory suggests that excessive revenue asymmetry reduces uncertainty of outcome (68, 69). The NBA mitigates this through redistributive mechanisms; however, it is contended that direct transplantation into Europe risks producing new asymmetries rather than resolving existing ones, given heterogeneous market conditions.

Partial franchise closure introduces a second-order problem: reduced permeability. From a viewpoint of opinion, permeability—not balance alone—is a critical variable, as it determines whether elite consolidation functions as an integrative platform or a structural barrier. Restricted mobility risks decoupling merit from access, thereby weakening developmental incentives across tiers.

Evaluation must therefore shift from static comparisons to dynamic system monitoring. Metrics such as the Herfindahl–Hirschman Index, revenue Gini coefficients, and talent migration flows should be institutionalised within governance audits. These are not simply analytical tools but governance instruments.

The central argument advanced here is that competitive balance cannot be treated as an isolated outcome variable. It is co-produced by redistribution design, access rules, and talent flows. If concentration erodes perceived fairness, legitimacy may decline irrespective of commercial success.

## Stakeholder embeddedness and legitimacy under hybrid governance

The cumulative consequences of hybrid governance ultimately depend on how its institutional tensions are distributed across stakeholders. This institutional arrangement constitutes a redistribution of power across interdependent stakeholders rather than a linear reform process. Drawing on stakeholder theory (70–73), this section advances the position that legitimacy will depend on maintaining alignment across athletes, coaches, clubs, and the broader ecosystem.

**Figure 1:**
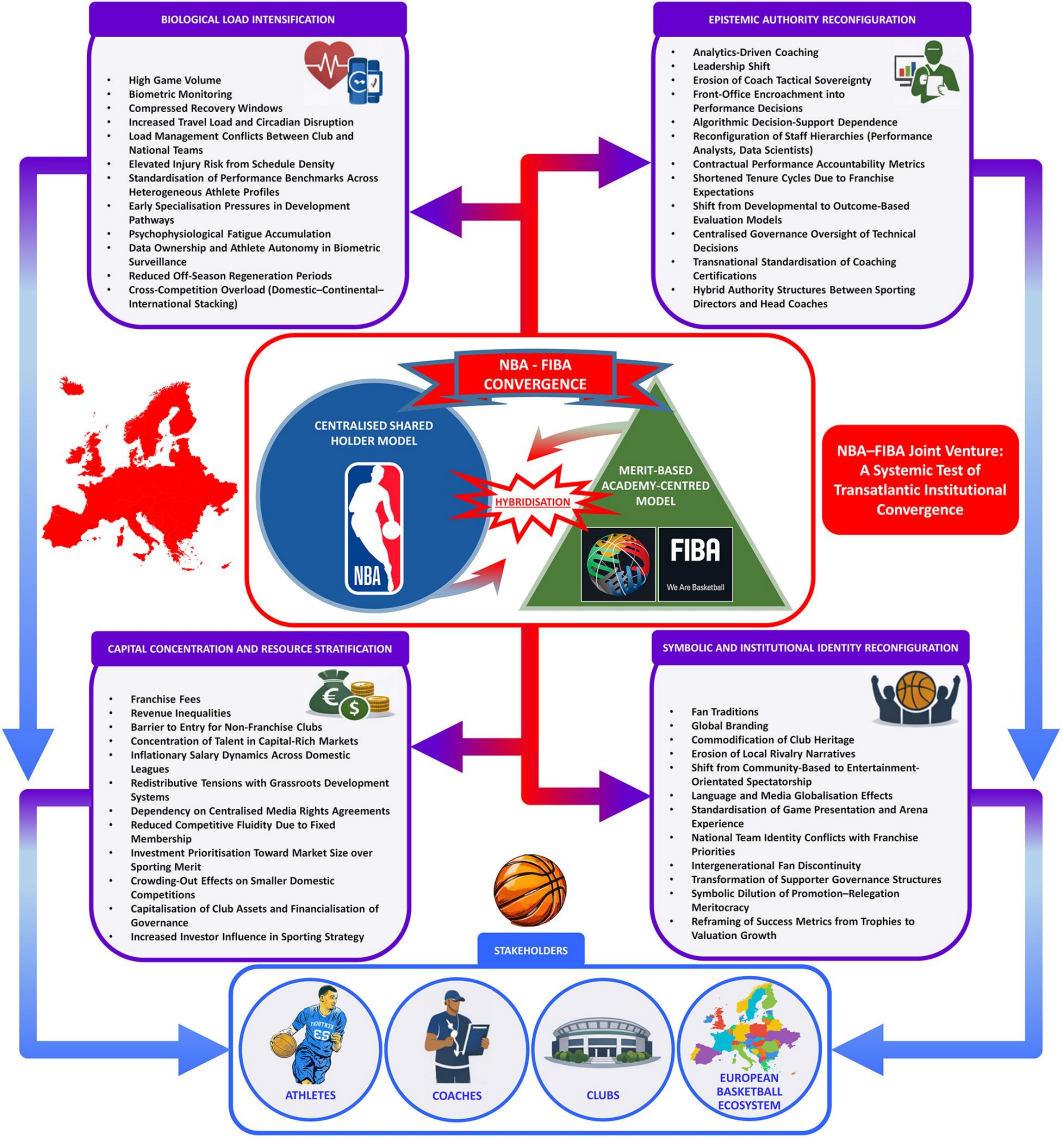
Stakeholder challenges in the NBA European basketball era. The figure conceptualises the collaboration between the NBA and the FIBA as a process of institutional hybridisation. It illustrates the convergence of a centralised, franchise-based commercial model with a meritocratic, pyramid-orientated European system. Four domains of governance friction—physiological demands, professional authority, financial redistribution, and cultural identity—mediate the impact on athletes, coaches, clubs, and the broader ecosystem. The model emphasises reciprocal adaptation across governance levels and suggests that the long-term legitimacy of NBA-Europe will depend on balancing commercial centralisation with developmental sustainability and merit-based access. [The figure was created by the authors using Microsoft 365, PowerPoint and Adobe Photoshop v.23. NBA and FIBA logos are registered trademarks of their respective organisations and are used for identification purposes only. No endorsement by these organisations is implied.].

The NBA–FIBA initiative can be understood as a hybrid institutional encounter in which friction emerges across four domains: physiology, authority, redistribution, and identity (Figure 1). Importantly, these are not independent axes but mutually reinforcing pressures (Figure 1).

For athletes, increased visibility is coupled with intensified workload and surveillance. It is argued that the key tension lies in the redefinition of autonomy under biometric governance, where optimisation may conflict with data ownership and labour rights (37). For coaches, legitimacy may become contingent. Authority shifts from hierarchical control to integrative capacity, requiring alignment between analytics, athlete welfare, and organisational strategy (38, 39, 53). Coaching power is therefore not reduced but reconstituted. For clubs, dual embeddedness becomes the defining challenge. Franchise inclusion offers economic stability but risks symbolic detachment from local supporters (59, 74). This creates a structural trade-off between global scalability and local legitimacy. At the ecosystem level, governance coherence may become fragile. Domestic leagues, national teams, and transnational competitions must negotiate authority and scheduling. Without coordination, hybrid governance risks generating systemic friction rather than integration (58).

The key proposition of this section is that stakeholder pressures are systemically coupled. Adjustments in one domain cascade across others. The success of hybrid governance therefore depends on maintaining reciprocal embeddedness rather than optimising individual stakeholder outcomes in isolation.

## Environmental accountability and carbon externalities in transnational competition

Environmental sustainability represents an additional test of whether commercial expansion can occur without generating new forms of institutional decoupling. The expansion of competitive geography under NBA–FIBA integration introduces an often underemphasised but structurally significant dimension: environmental externalities. A transnational league inherently increases aviation frequency, energy demand, and logistical complexity. From one perspective, NBA-Europe should be evaluated not only as a commercial or sporting system but as a carbon-intensive organisational architecture. Professional sport has already been identified as a contributor to aviation emissions and resource concentration (75–77).

The emerging league architecture amplifies these pressures through scheduling density and commercial expansion. Without mitigation, elevated emissions risk becoming structurally embedded. It is contended that sustainability cannot remain a peripheral commitment; it must be operationalised through measurable governance mechanisms. Life-cycle assessment and sport-specific carbon accounting should be integrated *ex ante*. Existing sustainability initiatives adopted by international sport organisations and major transnational sporting events (78, 79) demonstrate that environmental reporting can be embedded within governance frameworks rather than treated as a *post hoc* communication exercise. Within NBA-Europe, comparable governance indicators could include travel-related carbon emissions per team, arena energy intensity, renewable-energy utilisation, and scheduling efficiency. Integrating such indicators into routine governance reporting would enable environmental performance to be monitored alongside sporting and financial outcomes, thereby strengthening institutional accountability rather than relying on aspirational sustainability commitments. Without such frameworks, sustainability risks becoming symbolic rather than enforceable.

Infrastructure investment presents a parallel inflection point. Arena development must be evaluated through embodied carbon, energy integration, and long-term efficiency metrics. This represents a strategic choice: to reproduce high-consumption entertainment models or leverage sport as a platform for ecological innovation (80). The central claim is clear: environmental legitimacy will depend on whether sustainability is embedded within governance architecture rather than appended as an external objective.

## Methodological and structural limitations

As an opinion-based conceptual contribution, this article is intentionally interpretive rather than empirical. Its claims should therefore be understood as theoretically grounded propositions rather than testable conclusions. First, projections remain contingent on unresolved governance parameters. League structure, salary regulation, and scheduling design are not finalised. Accordingly, the analysis operates under informed assumptions rather than verified conditions. Second, the absence of precedent limits comparative inference. While partial analogues exist, no historical case replicates the institutional density of European basketball combined with NBA governance structures. Third, the framework assumes relative macro-level stability. Regulatory, economic, or geopolitical shifts could materially alter the trajectory of hybrid governance. Fourth, technological integration is treated as probable but not uniform. Variability in adoption may generate asymmetries not fully captured here. These limitations do not invalidate the analysis but define its scope: this article advances a structured interpretive lens designed to guide future empirical investigation.

## Future research directions: monitoring hybrid governance and systemic adaptation

The NBA–FIBA integration requires a deliberately constructed research agenda rather than retrospective analysis. The current phase offers a rare opportunity to define metrics before institutional path dependencies solidify. In this context, NBA-Europe should be treated as a longitudinal case of systemic transformation requiring coordinated, interdisciplinary monitoring. Six priority domains emerge: i) workload modelling should integrate travel, fatigue, and recovery into predictive threshold frameworks; ii) tactical evolution should be analysed through spatio-temporal tracking under hybrid rule conditions; iii) systemic equity requires continuous auditing through concentration and redistribution metrics; iv) supporter identity transformation should be examined longitudinally; v) technological governance demands validation and ethical scrutiny; and vi) comparative legal scholarship should examine compatibility between North American labour mechanisms and European Union competition frameworks. The overarching argument is that research must shift from descriptive analysis to embedded monitoring. The objective is not merely to observe change but to determine whether long-term institutional integration can occur without progressive structural misalignment.

## Conclusion: hybrid governance and the future of transatlantic basketball

The NBA–FIBA collaboration represents a structural experiment in hybrid governance rather than a conventional league expansion. Across all domains, the central challenge remains integration without decoupling. Performance systems, coaching authority, developmental pathways, competitive balance, stakeholder legitimacy, and environmental sustainability should therefore be understood as interconnected dimensions of a single governance problem rather than independent organisational challenges. They constitute an interconnected governance problem. The central conclusion is that the viability of NBA-Europe will depend less on commercial success than on systemic alignment. Franchise stability may enhance investment and visibility, but without redistribution, permeability, and cultural embeddedness, it risks institutional fragmentation. Hybrid governance can produce either adaptive integration or progressive decoupling. The determining factor will be whether governance architecture preserves interdependence across economic, developmental, and cultural domains. The coming decade will therefore not simply test a league model—it will test whether transatlantic convergence can generate a sustainable global configuration for basketball, or whether it will expose the limits of institutional hybridisation.
